# Phosphorylated Forms of STAT1, STAT3 and STAT5 Are Expressed in Proliferating but Not Involuted Infantile Hemangioma

**DOI:** 10.3389/fsurg.2018.00031

**Published:** 2018-04-19

**Authors:** Lucy Sulzberger, Elysia M. S. Tan, Paul F. Davis, Helen D. Brasch, Swee T. Tan, Tinte Itinteang

**Affiliations:** ^1^Gillies McIndoe Research Institute, Wellington, New Zealand; ^2^Centre for the Study and Treatment of Vascular Birthmarks, Wellington Regional Plastic, Maxillofacial and Burns Unit, Hutt Hospital, Wellington, New Zealand

**Keywords:** STAT1, STAT3, STAT5, phosphorylated, infantile hemangioma, JAK/STAT pathway

## Abstract

We have recently demonstrated the expression of embryonic stem cell markers on the endothelium of infantile hemangioma, a functional hemogenic endothelium with the capacity for primitive erythropoiesis *in vitro*. Despite recent work characterizing stem cells within proliferating infantile hemangioma, the expression of STAT proteins, well documented for their roles in stem cell signaling, has not been investigated. 3,3-Diaminobenzidine and immunofluorescence immunohistochemical staining revealed expression of pSTAT1, pSTAT3 and pSTAT5 in proliferating infantile hemangioma samples with the strongest expression of pSTAT3. There was reduced expression of these pSTAT proteins in the involuted infantile hemangioma samples. Western blotting confirmed the identification of all these three proteins in proliferating infantile hemangioma. It is therefore not surprising that the phosphorylated/activated forms of these proteins are relatively abundantly expressed in proliferating, in comparison to involuted infantile hemangioma samples. We speculate that the reduced STAT activation, as infantile hemangioma involutes, is a reflection of the depletion of the abundant stem cells within proliferating infantile hemangioma, as the lesion involutes.

## Introduction

Infantile hemangioma (IH), the most common tumor of infancy, affects up to 10% of infants with a predilection for female, Caucasian and premature infants (1–3). It typically undergoes rapid growth during infancy followed by a spontaneous gradual involution over 1–10 years, often leaving a fibro-fatty residuum (4). The understanding of this enigmatic condition has been advanced by the appreciation of the role of stem cells within the endothelium and the interstitium of proliferating IH (5–7). The eventual laydown of fibro-fatty residuum in involuted IH has been attributed to the presence of an IH mesenchymal stem cell (IHMSC) population, a distinct non-clonal and downstream population of the IH stem cell (IHSC) precursor within proliferating IH (8).

We have recently documented the expression of embryonic stem cell (ESC) markers OCT4, signal transducer and activator of transcription 3 (STAT3) and stage-specific embryonic antigen-4 (SSEA-4) on the endothelium of proliferating IH (6). The endothelium of proliferating IH has also been shown to be a functional hemogenic endothelium (HE) that expresses erythropoietin receptor (EPOR) and hemoglobin ζ chain with a capacity for primitive erythropoiesis *in vitro *(9, 10).

The STAT family acts as intracellular messengers in a variety of roles in stem cells and more specifically the hematopoietic stem cell (HSC) populations, including precursor expansion (11). Latent STATs are activated by Janus-Kinase mediated phosphorylation in the cytosol following ligand binding at cell surface receptors (12). Once phosphorylated, STAT (pSTAT) proteins dimerize and translocate to the nucleus to modulate gene transcription (12).

In the context of hematopoiesis, STAT1 signaling via IFN-α stimulation controls dormant HSC’s entry into the cell cycle (11) whilst STAT1 signaling downstream of IFN-γ promotes the expansion of HSCs (13). STAT3 is a key mediator of pluripotent cell maintenance and is a regulator of the NANOG-OCT4 pathway (14). We have previously shown the expression of the non-phosphorylated form of STAT3 on the endothelium and cells within the interstitium of proliferating IH, inferring its role in the stem cells within proliferating IH, although the localization of the phosphorylated/activated form of this transcription factor remains to be determined (3). STAT5 acts as a downstream messenger of EPOR in both primitive and definitive erythropoiesis and its absence is embryonically fatal (15).

Despite recent advances in the characterization of the stem cell populations within proliferating IH (6, 16) the expression of pSTAT1, pSTAT3 and pSTAT5 has not been investigated in this tumor. We hypothesize their presence within the stem cell populations of proliferating IH. This study aimed to examine the expression of these phosphorylated/activated transcription factors within IH.

## Material and Methods

### Tissue Samples

Eight proliferating and eight involuted IH samples obtained from patients undergoing surgical excision of IH were used for this study, which was approved by the Central Regional Health and Disability Ethics Committee (ref. no. 13CEN130). Written consents were obtained from all participants.

### Immunohistochemical Staining

4µm-thick formalin-fixed paraffin-embedded sections of proliferating (*n* = 8) and involuted (*n* = 8) IH underwent 3,3-diaminobenzidine (DAB) IHC and immunofluorescence (IF) IHC staining on a Leica ASP200S Autostainer (Leica, Nussloch, Germany), using the Bond Polymer Refine Ready-to-use Detection Kit (Leica) for DAB staining. Slides were prepared for auto-staining by dewaxing and heat induced epitope retrieval using Bond Epitope Retrievals (Leica).

The primary antibodies used were pSTAT1 (1:800; cat# SC-135648, Santa Cruz Biotechnology, Dallas, TX, USA), pSTAT3 (1:400; cat# D3A7, Cell Signaling Technology, Danvers, MA, USA), pSTAT5 (1:400; cat# C7IE5, Cell Signaling Technology), GLUT-1 (1:200; cat# 355A-14, Cell Marque, Rocklin, CA, USA) and CD34 (ready-to-use, cat# PA0212, Leica). Primary antibodies for IF IHC staining were detected using Vectafluor Excel anti-rabbit 594 (ready-to-use; cat# VEDK-1594, Vector Laboratories, Burlingame, CA, USA) and Alexa Fluor anti-mouse 488 (1:500; cat# A21202, Life Technologies, Carlsbad, CA, USA).

DAB IHC-stained slides were counterstained with hematoxylin prior to cover slipping and mounted using Surgipath Micromount mounting media (Leica). IF IHC-stained slides were mounted using Vectashield hardset mounting medium with 4', 6-diamidino-2-phenylindole (Vector Laboratories). Human tonsillar tissue was used as a positive control for pSTAT1, pSTAT3 and pSTAT5 and human placental tissue was used for CD34.

### Image Capture

DAB IHC-stained slides were viewed and the images were captured using an Olympus BX53 light microscope fitted with an Olympus DP21 digital camera (Tokyo, Japan). IF IHC-stained slides were viewed and the images were captured using an Olympus FV1200 biological confocal laser-scanning microscope (Tokyo, Japan).

### Western Blotting

One snap-frozen proliferating and one involuted IH tissue samples from the original cohort of patients used for DAB IHC staining were used for Western blotting (WB). These tissue samples were processed as previously described (3) and transferred to nitrocellulose membranes (Thermo Scientific) using an iBlot 2 (Thermo Scientific). The membranes were blocked for 90 min at 4°C in 1x iBind™ Flex Solution (Thermo Scientific) and probed using an iBind™ Flex device (Thermo Scientific) with the following primary antibodies: pSTAT1 (1:1,000; cat# 9167, Cell Signaling Technology, Danvers, MA, USA), pSTAT3 (1:1,000; cat# 9145, Cell Signaling Technology), pSTAT5 (1:1000; cat# 9314, Cell Signaling Technology) and β-actin (1:2,000; cat# ab8226, Thermo Scientific). Detection and imaging of the blots were undertaken as routinely done in our laboratory (3). Human tonsil, mouse lung, and human liver total protein extracts were used as the positive controls for pSTAT1, pSTAT3, and pSTAT5, respectively.

## Results

### Histochemistry and 3,3-Diaminobenzidine Immunohistochemical Staining

Proliferating IH showed plump, proliferating endothelial cells, organized into lobules with tiny lumina (Figure S1A). This cellular parenchyma was replaced by loose fibrofatty tissue in involuted IH (Figure S1B). All IH samples used for this study were confirmed to be IH by their expression of GLUT-1 (data not shown). DAB IHC staining demonstrated strong nuclear and cytoplasmic expression of pSTAT1 on the endothelial and pericyte layers, and cells within the interstitium in proliferating IH (Figure S2A, brown), but not in involuted IH (Figure S2B, brown) lesions. Strong nuclear and cytoplasmic expression of pSTAT3 was observed in proliferating IH (Figure S2C, brown) with reduced expression in involuted IH (Figure S2D, brown) lesions. pSTAT5 was localized to the nuclei of the endothelial cells in proliferating IH (Figure S2E, brown) and was absent in involuted IH (Figure S2F, brown) samples.

### Immunofluorescence Immunohistochemical Staining

**Figure 1:**
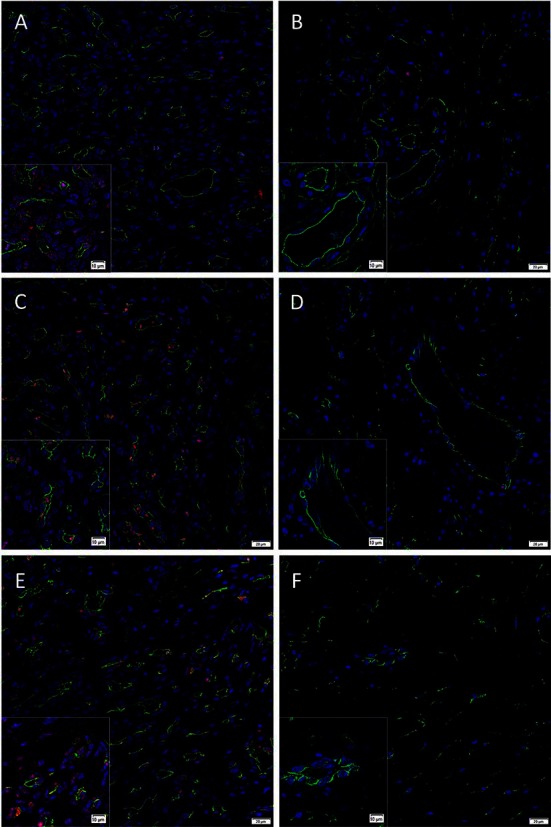
Representative immunofluorescence immunohistochemical*-*stained sections of proliferating **(A, C, E)** and involuted **(B, D, F)** IH samples demonstrating expression of pSTAT1 (**A,B**, red), pSTAT3 **(C, D**, red**)** and pSTAT5 **(E, F**, red**)**, double-stained with CD34 **(A–F**, green**)**. There was reduced or no expression of these proteins in involuted IH samples **(B, D and F)**. Cell nuclei were counter-stained with 4′, 6-diamidino-2-phenylindole (blue). Scale bars: 20 µm. Inserts: magnified views.

To determine the localization of the cells expressing the pSTAT1, pSTAT3 and pSTAT5 proteins we performed IF IHC co-staining of these markers with the endothelial cell marker CD34 (Figure 1A–F, green) in proliferating (Figure 1A,C,E) and involuted (Figure 1B,D,F) IH samples. This demonstrated the expression of pSTAT1 (Figure 1A,B, red) by cells on the endothelium, the pericyte layer and the interstitium of proliferating IH (Figure 1A), but not involuted (Figure 1B) IH lesions. Abundant nuclear staining of pSTAT3 (Figure 1C,D, red) was demonstrated on the endothelial cells and cells within the interstitium in proliferating (Figure 1C), and very few endothelial cells within involuted (Figure 1D) IH lesions. pSTAT5 (Figure 1E,F, red) was expressed mostly by cells on the endothelium and cells within the interstitium, away from the CD34 endothelium in proliferating IH (Figure 1E), but not in involuted IH (Figure 1F) lesions.

### Western Blotting

**Figure 2:**
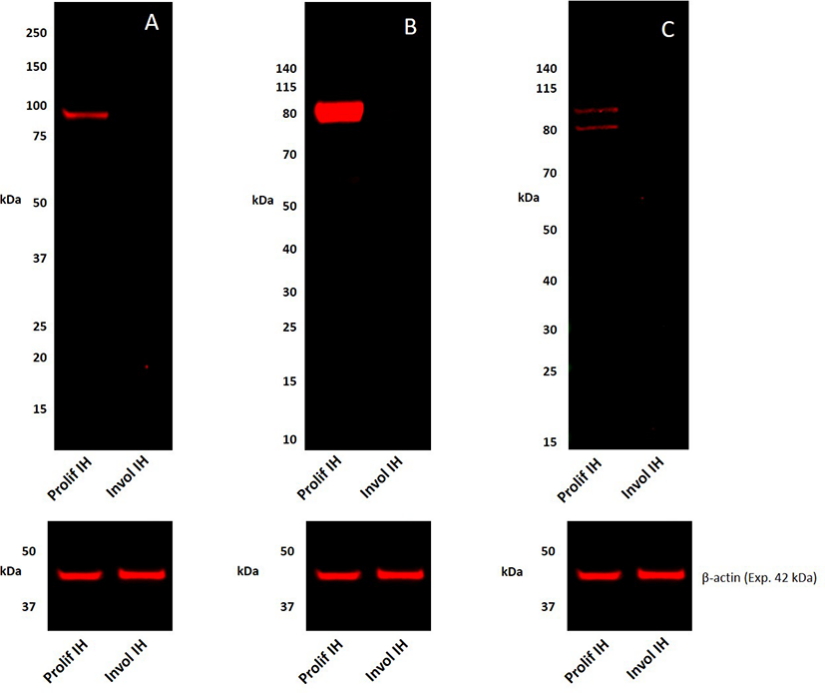
Representative 1DE Western blot images of separated total protein extracts of a proliferating and an involuted IH samples probed for pSTAT1 **(A)**, pSTAT3 **(B)**, and pSTAT5 **(C)** and detected with HRP conjugated goat anti-rabbit secondary antibody. β-actin was used as the loading control and detected using Alexa® 647 rabbit anti-mouse secondary antibody **(A–C)**.

To confirm the protein identification by IHC staining, WB analyses of tissue lysates of a proliferating sample and an involuted IH sample for the presence of pSTAT1, pSTAT3, and pSTAT5 were performed. pSTAT1 was detected as a single band at the expected 91 kDa (17) in proliferating but was undetectable in the involuted IH extract (Figure 2A) which was in agreement with the human tonsil positive control sample (Figure S3A). pSTAT3 was detected as two thick bands at approximately 86 and 79 kDa in the proliferating but was undetectable in the involuted IH extract (Figure 2B). Similar bands were also detected in the mouse lung positive control sample (Figure S3B), potentially corresponding to the α- and β-pSTAT3 isoforms, respectively (18). pSTAT5 was detected in the proliferating but was undetectable in the involuted IH extracts (Figure 2C), as two bands at approximately 80 kDa and 90 kDa as previously described (19), consistent with the human liver positive control sample (Figure S3C). Approximate equivalent protein loading across the samples was confirmed using β-actin (Figure 2).

## Discussion

The demonstration of the expression of pSTAT1, pSTAT3 and pSTAT5, and their localization in IH, is novel. The observation of phosphorylated/activated forms of these proteins being abundantly expressed in proliferating and markedly reduced or not expressed in involuted IH samples, may be due to down-regulation of STAT activation, associated with depletion of the relatively abundant stem cells in proliferating IH, as the lesion involutes (5).

Down-regulation of pSTAT1 as IH involutes, is likely to be related to stem cell activity with mast cell (20) and macrophage (21) populations previously identified within IH, highlighting a potential local origin of the STAT1 activators IFN-α and IFN-γ. The putative role of pSTAT1 in IHSC maintenance may also correlate with terminal differentiation of IHMSC as the lesion involutes with adipogenesis and cessation of hematopoietic activity, and the apparent absence of pSTAT1 in involuted IH lesions, as presented in this report. Our finding of the presence of pSTAT1 by IHC staining is supported by the identification of the appropriate sized protein on WB of the proliferating but not involuted IH samples.

pSTAT3 is the most abundantly expressed of the STAT proteins investigated in this study. STAT3 signaling is crucial in stem cell maintenance (22) and the expression of pSTAT3 beyond the HE of proliferating IH correlates with the demonstration of IHMSCs in the pericyte and interstitial cell populations of IH. Our finding of the presence of pSTAT3 by IHC staining is also, in-part, supported by the identification of the appropriate protein on WB of the proliferating but not involuted IH samples.

Components of the renin-angiotensin system (RAS) have been reported to be expressed by proliferating IH (23, 24). Widespread activation of STAT3 in IH may parallel ligand binding to angiotensin II receptor 2 with angiotensin II, a known inducer of STAT3 activation (25). The proposed role of the RAS in regulating the HE of proliferating IH underscores the spontaneous and accelerated involution of proliferating IH induced by β-blockers (2) and ACE inhibitors (26). The clinical observation of accelerated involution of IH induced by these RAS modulators may be a result of reduced STAT3 signaling leading to the loss of stem cell maintenance. 

pSTAT5 expression within proliferating IH is not surprising in light of the demonstration of the expression of EPOR on the HE of proliferating IH (9). This study shows nuclear staining of pSTAT5 in the endothelial cells and cells in the interstitium of proliferating IH. A protein band at the expected size for pSTAT5 identified by WB, consistent with the positive control, in the proliferating but not involuted IH sample, supports the finding of IHC staining. As pSTAT5 is essential for primitive hematopoiesis and signaling downstream of EPOR (15) its activity further supports the presence of a functional HE within proliferating IH. This is not entirely unexpected given the known plasticity of stem cells from a hemogenic endothelial phenotype to undergo hematopoiesis (27).

This report presents novel findings of the expression of pSTAT1, pSTAT3 and pSTAT5 within proliferating IH. We speculate that expression of pSTAT1, pSTAT3 and pSTAT5 in cells of the endothelium and the interstitium of proliferating IH, may represent their involvement in putative stem cell signaling by both these populations, although further work is needed to clarify this. We propose that increased expression of these phosphorylated/activated proteins during proliferation and their reduction or absence during involution may reflect their involvement in stem cell maintenance, although conclusive proof of this is beyond the scope of this work.

There are increasing reports of the role of stem cells in the pathogenesis of IH and this supported by the confirmed expression of markers such as alkaline phosphatase and CD133 on the endothelial cells of proliferating IH (28). The demonstration of the expression of aforementioned pSTAT proteins investigated in this report on the same proliferating IH endothelium, supports a crucial role for their involvement in the signaling pathways of stem cells in this tumor.

### Take Home Messages

1. The phosphorylated/activated forms of STAT1, STAT3 and STAT5 are present in the endothelium and cells in the interstitium of proliferating infantile hemangioma.
2. The phosphorylated/activated forms of STAT1, STAT3 and STAT5 are reduced or absent in involuted infantile hemangioma.
3. This finding suggests roles for STAT1, STAT3 and STAT5 in the biology of stem cells within infantile hemangioma.

## Disclaimer

Aspects of this work was presented at the Medical Science Congress, Queenstown, New Zealand, August 25–27, 2014; the New Zealand Association of Plastic Surgeons’ Annual Scientific Meeting, Queenstown, New Zealand, September 5–7, 2014; and the Plastic Surgery Congress, Brisbane, Australia, May 7–10, 2015. This work was also part of LS’s Bachelor of Medical Science thesis “The Activity of the JAK-STAT Pathway in Infantile Hemangioma and the Hemogenic Potential of Infantile Hemangioma Explant Derived Cells”, University of Otago, New Zealand.

## Ethics Statement

This study was approved by the Central Regional Health and Disability Ethics Committee (ref. no. 13CEN130). Written consents were obtained from all participants.

## Author Contributions

LS, TI and ST came up with the study hypothesis and designed the study. LS, ET, TI, HB and ST interpreted the IHC staining results. LS analyzed the Western blotting data. LS, ET, PD, TI and ST drafted the manuscript. All authors approved the manuscript.

## Conflict of Interest Statement

The authors declare that the research was conducted in the absence of any commercial or financial relationships that could be construed as a potential conflict of interest.

## Supplementary material

Figure S1 A representative H&E sections of a proliferating IH showing plump endothelial cells, organized into lobules with tiny lumens **(A)**. Involuted IH with interlobular and intralobular fibrosis and few thick-walled channels **(B)**. Original magnification: 400×.

Figure S2 Representative 3,3-diaminobenzidine immunohistochemical stained sections of proliferating **(A, C** and **E)** and involuted **(A, D** and **F)** IH samples showing expression of pSTAT1 **(A** and **B**, brown**)**, pSTAT3 **(C and D**, brown**)** and pSTAT5 **(E**and **F**, brown**)**. There was reduced or no expression of these proteins in involuted IH samples **(B, D,** and **F)**. Cell nuclei were counter-stained with hematoxylin (blue). Original magnification: 400×.

Figure S3 Representative Western blot images of 1DE separated total protein extracts of positive controls human tonsil demonstrating the presence of pSTAT1 detected as a single band at 91 kDa **(A)**, mouse lung demonstrating the presence of pSTAT3 detected as two thick bands at approximately 86 and 79 kDa **(B)** and human liver demonstrating the presence of pSTAT5 as two bands at approximately 80 and 90 kDa **(C)**.
