# Supplementary figures and images for: Phosphorylated Forms of STAT1, STAT3 and STAT5 Are Expressed in Proliferating but Not Involuted Infantile Hemangioma
Source: Front Surg. 2018 Apr 19;5:31. doi: 10.3389/fsurg.2018.00031 (PMC5917091; doi:10.3389/fsurg.2018.00031)

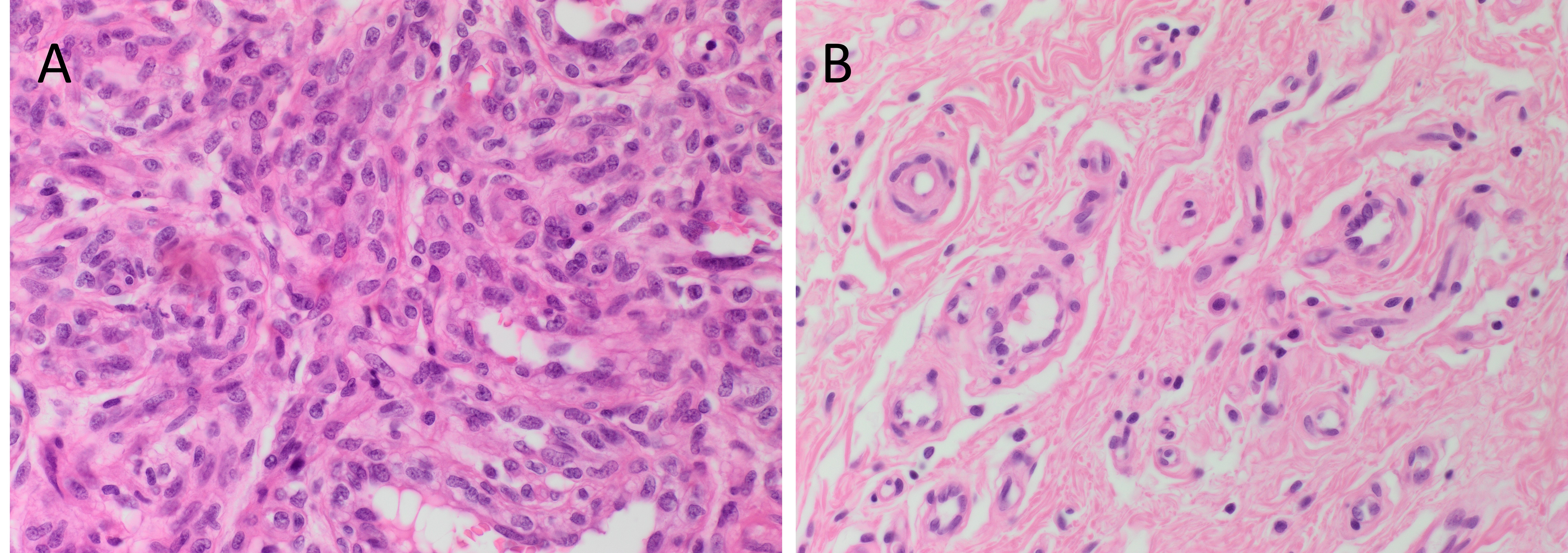

Supplement: Figure S1 — A representative H&E sections of a proliferating IH showing plump endothelial cells, organized into lobules with tiny lumens (A). Involuted IH with interlobular and intralobular fibrosis and few thick-walled channels (B). Original magnification: 400×. [file Image1.JPEG]

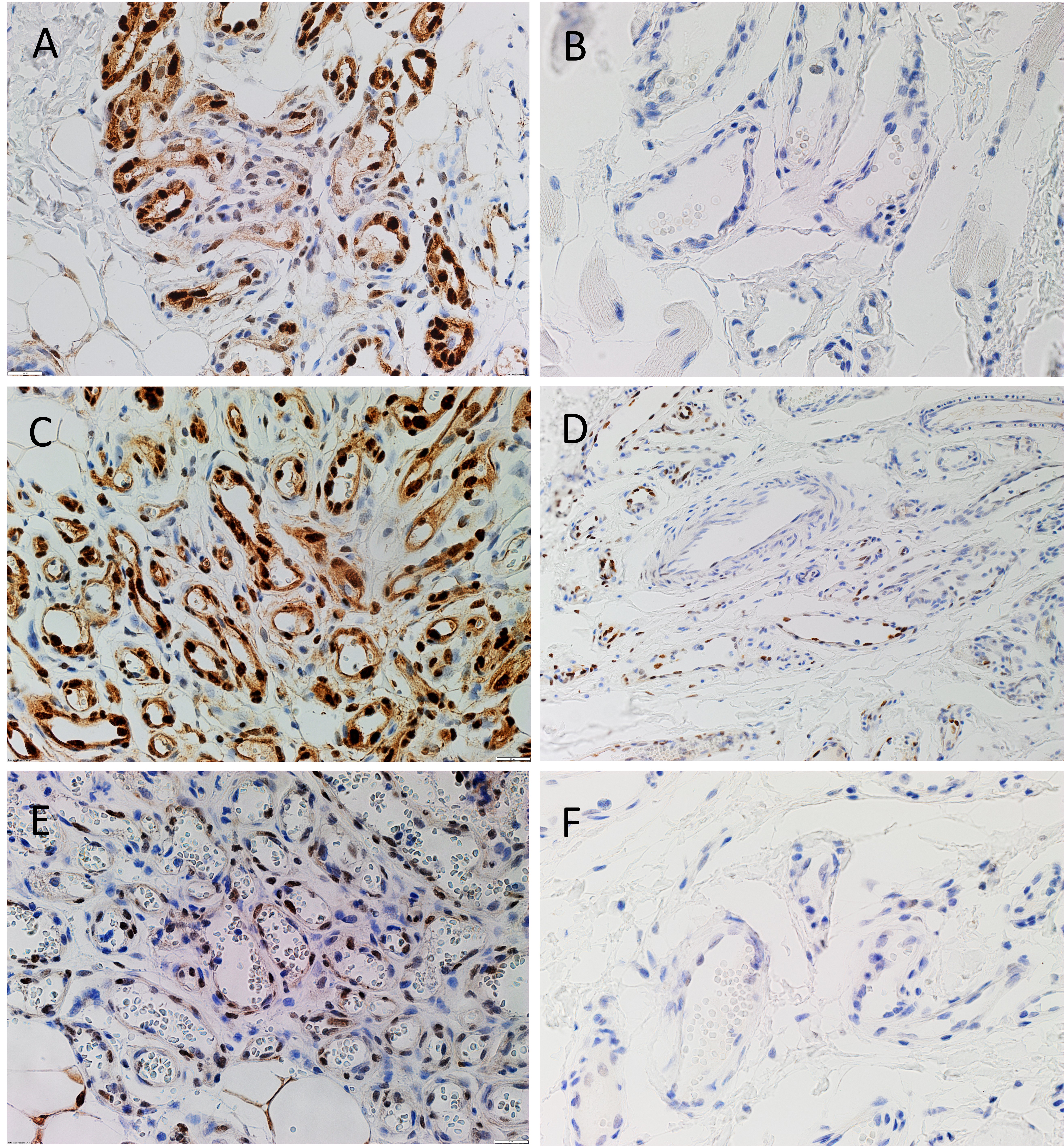

Supplement: Figure S2 — Representative 3,3-diaminobenzidine immunohistochemical stained sections of proliferating (A, C and E) and involuted (A, D and F) IH samples showing expression of pSTAT1 (A and B, brown), pSTAT3 (C and D, brown) and pSTAT5 (Eand F, brown). There was reduced or no expression of these proteins in involuted IH samples (B, D, and F). Cell nuclei were counter-stained with hematoxylin (blue). Original magnification: 400×. [file Image2.JPEG]

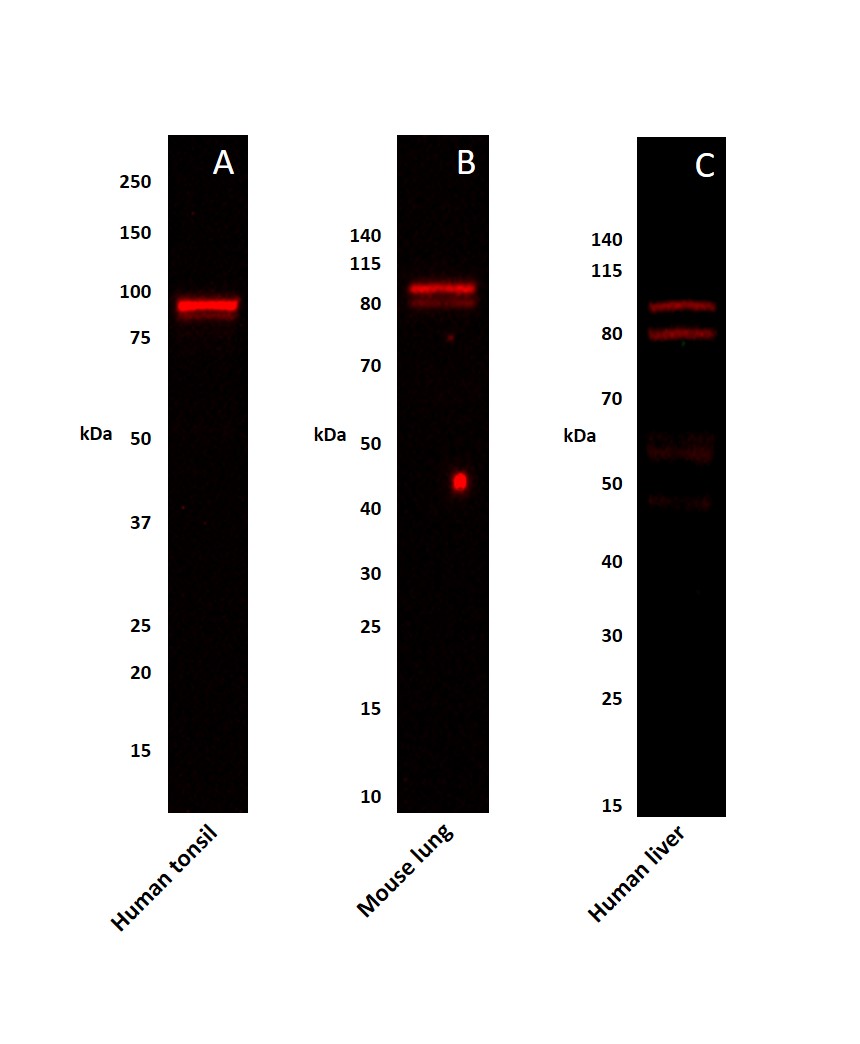

Supplement: Figure S3 — Representative Western blot images of 1DE separated total protein extracts of positive controls human tonsil demonstrating the presence of pSTAT1 detected as a single band at 91 kDa (A), mouse lung demonstrating the presence of pSTAT3 detected as two thick bands at approximately 86 and 79 kDa (B) and human liver demonstrating the presence of pSTAT5 as two bands at approximately 80 and 90 kDa (C). [file Image3.JPEG]
